# Laser processing of thin-film multilayer structures: comparison between a 3D thermal model and experimental results

**DOI:** 10.3762/bjnano.8.176

**Published:** 2017-08-24

**Authors:** Babak B Naghshine, Amirkianoosh Kiani

**Affiliations:** 1 Silicon Hall: Laser Micro/Nano Fabrication Facility, Department of Mechanical Engineering, University of New Brunswick, NB, Canada; 2 Department of Automotive, Mechanical and Manufacturing Engineering, University of Ontario Institute of Technology (UOIT), ON, Canada

**Keywords:** 3D transient modelling, heat transfer, laser materials processing, nanosecond pulses, silicon, thin-film

## Abstract

In this research, a numerical model is introduced for simulation of laser processing of thin film multilayer structures, to predict the temperature and ablated area for a set of laser parameters including average power and repetition rate. Different thin-films on Si substrate were processed by nanosecond Nd:YAG laser pulses and the experimental and numerical results were compared to each other. The results show that applying a thin film on the surface can completely change the temperature field and vary the shape of the heat affected zone. The findings of this paper can have many potential applications including patterning the cell growth for biomedical applications and controlling the grain size in fabrication of polycrystalline silicon (poly-Si) thin-film transistors (TFTs).

## Introduction

Laser processing of thin-film multilayer structures has been studied since the early 1990s [1]. However, the field has been largely neglected by researchers and there have been very few studies in this area [2–4]. On the other hand, it has many interesting applications that can introduce new possibilities to the art of laser processing. One of the most interesting applications is the fabrication of polycrystalline silicon (poly-Si) thin-film transistors (TFTs). In this method, a thin film of amorphous silicon (a-Si) is deposited on a glass substrate, where it is processed by a UV laser beam. Since a-Si has a very high absorption at UV spectrum, it can be recrystallized into poly-Si by the laser beam without causing any considerable temperature change in the substrate [5–7]. There are also various laser doping techniques such as spin coating the Si layer with a phosphorus- or boron-containing liquid before laser processing the surface for n- and p-type TFTs respectively [8]. Another important application is mask repairing for MEMS/NEMS device fabrication [4]. There are also many potential applications in biomedical engineering. It’s been proven that laser processing a metallic surface can significantly improve the biocompatibility in the ablated area and living cells can adhere better to the surface [9–13] whereas, the heat affected zone becomes less biocompatible and cells will avoid that area [14]. Therefore, the growing pattern of the cells can be controlled by laser processing of the surface which has many potential applications in biomedical engineering. As will be shown in this paper, the shape and size of these zones can be controlled by applying a thin-film layer on the surface. Altering the electrical properties of the surface for biosensor fabrication is one of the applications that has not been fully explored yet.

Modelling this process can be helpful in many ways. For instance, controlling the grain size of the resulting poly-Si layer is vital in the process of fabricating poly-Si TFTs [6–7]. This can be only obtained by controlling the temperature and cooling rate of the thin film during laser processing. As the process occurs over a very short time it’s difficult to measure the temperature, and a proper thermal model can be the most useful tool for controlling the temperature. Another possible application is finding the best laser parameters and layer thickness for controlling the size of heat affected zone and ablated zone for patterning the cell, as mentioned in the previous paragraph.

Despite the necessity for thermal modelling of this process, there have been very few efforts in this area so far. In previous works, only simple one-dimensional models were used to calculate the temperature of different thin-film structures without considering the phase change [4,15].

There are many models for laser processing of bulk materials [16–21]. There are some analytical solutions to the one- or two-dimensional heat conduction equation to predict the temperature. An example can be found in the work of Hendow and Shakir, who solved the 2D equation to calculate the temperature field after irradiating the surface with two consecutive pulses [16]. Another example is the work of Chen et al. who solved the same equation for repetitive laser pulses [17]. There are also many numerical models that provide us with much more information about the process. Weidmann et al. carried out a numerical analysis by solving the 2D equation. They used Arhenius equation for estimation of the speed of ablation to predict the ablated area [18]. Sinha numerically solved the 2D heat equation assuming phase changes by changing the size of computational cells during ablation [19]. In some models plasma shielding was taken into account as an effective factor. An example can be seen in the work of Marla et al. who solved the 1D equation for temperature-dependent properties [20].

In this paper, a transient 3D model, which has been previously proven capable of precisely predicting the temperature and ablation zone for a bulk material [21], has been modified and used for a thin-film multilayer structure. In this model, the physical and thermal properties are assumed to be a function of temperature and space; plasma shielding and phase change are taken into account. The temperature field and ablated area will be for Si substrates that are coated with different thin-film layers at various laser parameters. Finally, a comparison will be made between the numerical and experimental results.

## Results and Discussion

The samples were processed with different laser parameters (power and frequency) and the hole profiles were captured using the 3D optical profiler from at least ten different points for each sample.

### Laser processing of Si substrate (bulk material)

The melting and boiling points of silicon are 1414 °C and 3538 °C respectively and the heats of fusion and vaporization are 1788 and 13637 kJ/kg [22]. After introducing all the physical properties and the plasma absorption of the silicon, the numerical analysis was carried out and calculated and measured profiles were compared to each other.

**Figure 1:**
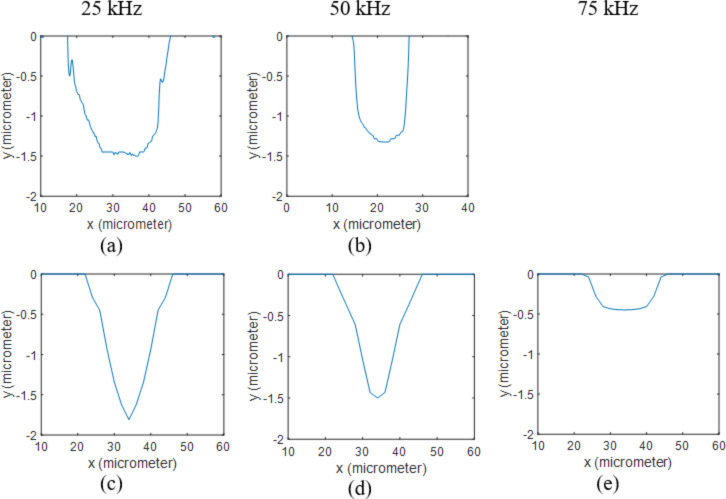
2D profiles of uncoated Si samples processed at 5 W. Results obtained from measurements when using (a) 25 kHz and (b) 50 kHz are compared to the ones calculated through the numerical model at (c) 25 kHz, (d) 50 kHz and (e) 75 kHz.

As can be seen in Table 1, the numerical and experimental results are in good agreement. The results for varying frequencies at 5 W are presented in Figure 1. The holes on the surface of the specimen that was processed at 75 kHz and 5 W were very shallow and it was difficult to capture a precise profile using the 3D optical profiler. This was mainly due to the noise captured by the equipment (there were some fluctuations around 0.2–0.3 μm on their surfaces). However, the 2D images of the sample’s surface verify the formation of these shallow holes on the surface. This is in agreement with the calculated depth (0.4 μm).

**Table 1:** Measured and calculated data for the ablation cross-section for uncoated Si samples.

Power (W)	Frequency (kHz)	Averaged measured depth (μm)	Calculated depth (μm)	Accuracy (%)

5	25	1.6	1.8	87.5
5	50	1.4	1.5	92.9
5	75	–	0.4	–
10	50	2.2	2.1	95.6
15	50	1.9	1.9	100

**Figure 2:**
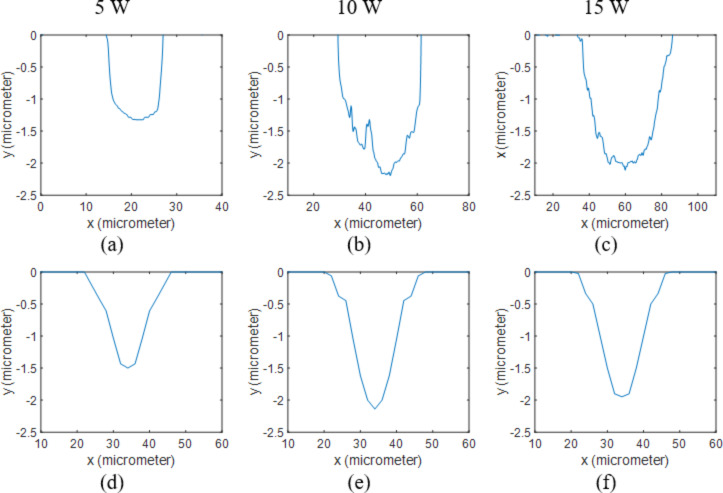
2D profiles of uncoated Si samples processed at 50 kHz. Results obtained from measurements when using (a) 5 W, (b) 10 W and (c) 15 W are compared to the ones calculated through the numerical model at (d) 5 W, (e) 10 W and (f) 15 W.

Figure 2 shows the profiles for varying laser power at the repetition rate of 50 kHz. The hole profiles were successfully calculated and are in accordance with experimental results.

The diameters of the holes at low frequencies and high powers are slightly larger in the experimental results. The laser-driven shock wave and the flow of the molten silicon, which were not considered in the model, can explain the smaller calculated hole diameter.

**Figure 3:**
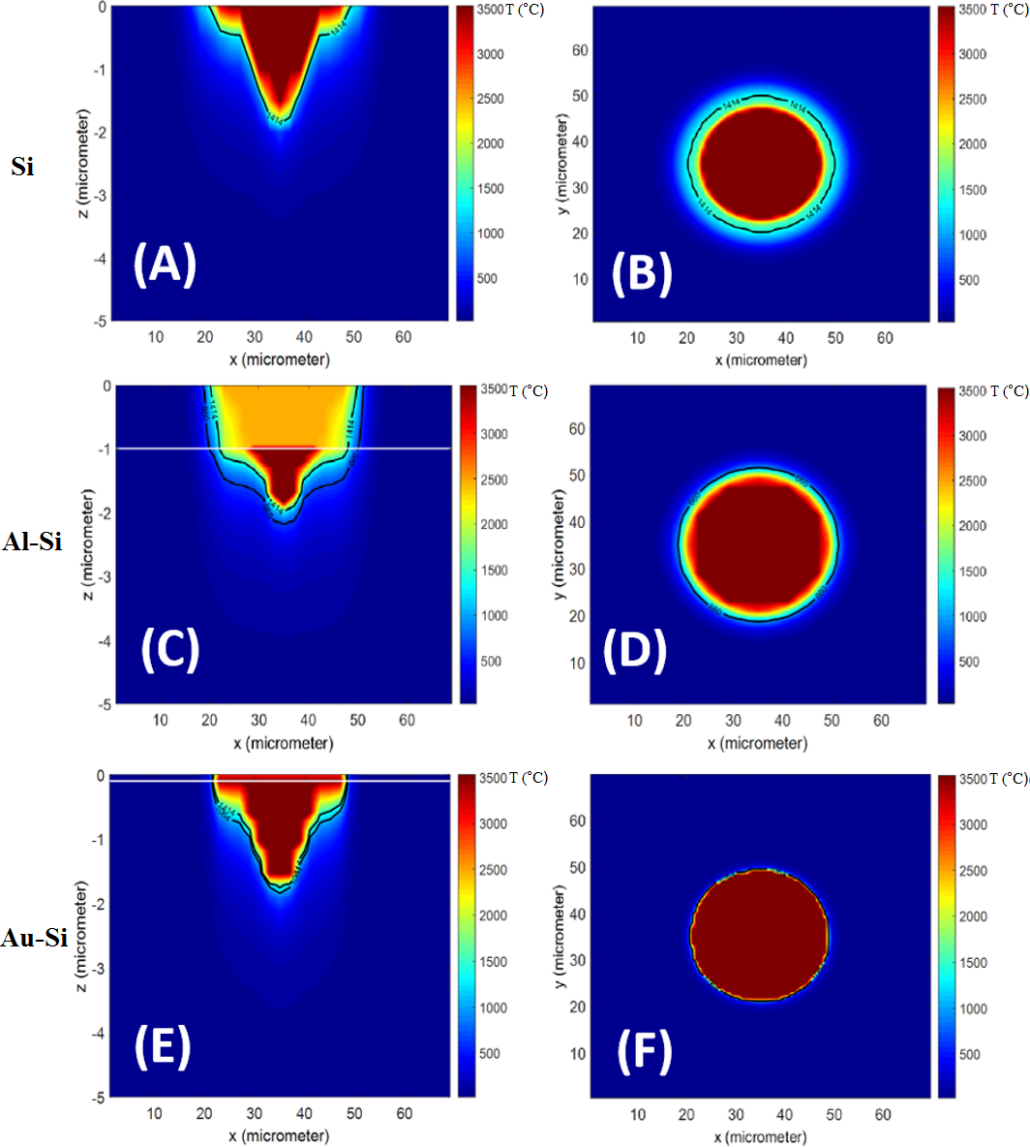
Temperature contours obtained from the numerical model at the end of the pulse at 50 kHz and 5 W for (a) cross-section of uncoated Si, (b) surface of uncoated Si, (c) cross-section of Al coated Si (d) surface of Al coated Si, (e) cross-section of Au coated Si, (f) surface of Au coated Si. The melting points of the materials are depicted by black lines and the interface between the layers is shown by white lines.

Figure 3 shows the temperature contour at the end of the pulse on the surface and cross-section of the silicon sheet for a repetition rate of 50 kHz and a power of 5 W. The melting point is highlighted and shows a molten zone formed around the ablated area. The pressure difference caused by the shock wave can push away the molten material and make both molten and ablated zones slightly larger. At low frequencies and high powers, the peak power of the laser pulses would be larger, which results in a stronger shock wave. The fluid dynamics of the molten material is not in the scope of this paper and was not considered in the model.

### Laser processing of Al-Si thin-film substrate

**Figure 4:**
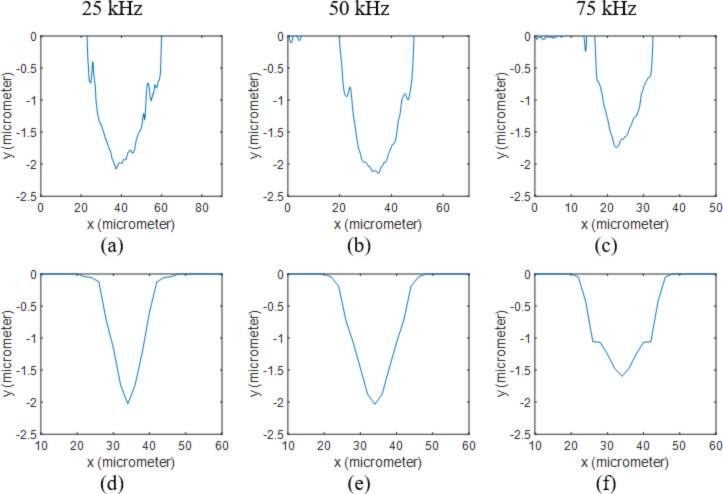
2D profiles of Al coated Si samples processed at 5 W. Results obtained from measurements when using (a) 25 kHz, (b) 50 kHz and (c) 75 kHz are compared to the ones calculated through the numerical model at (d) 25 kHz, (e) 50 kHz and (f) 75 kHz.

**Figure 5:**
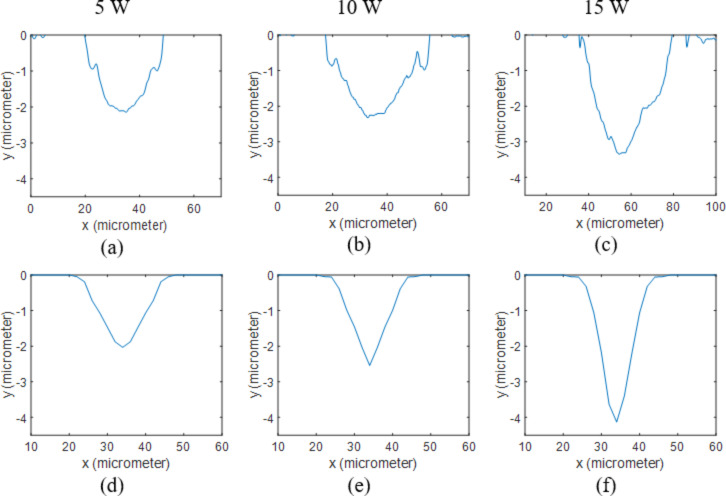
2D profiles of Al coated Si samples processed at 50 kHz. Results obtained from measurements when using (a) 5 W, (b) 10 W and (c) 15 W are compared to the ones calculated through the numerical model at (d) 5 W, (e) 10 W and (f) 15 W.

The melting and boiling points of aluminum are 660 °C and 2470 °C respectively and aluminum has a latent heat of fusion of 397 kJ/kg and a latent heat of vaporization of 10,800 kJ/kg [22]. The measured and calculated ablation depths are presented in Table 2. The surface profiles at different frequencies and 5 W are shown in Figure 4. By applying a thin aluminum film to the surface, the ablation depth was significantly increased at 75 kHz compared to bare silicon samples. This can be explained by the smaller latent heat of vaporization and lower boiling temperature of aluminum, which means more material can be ablated using the same amount of energy. This can be further verified by the deeper holes at 25 and 50 kHz. The other reason is the lower plasma absorption of aluminum compared to silicon based on the calculated plasma absorption functions. Since the single pulses are studied here, the processing time is extremely short and the material plume mainly contains the particles of the material of the thin film, which results in completely different plasma absorption functions for the three samples. Similar conclusions can be made from Figure 5 for various powers, and the holes are generally deeper in the aluminum coated samples. Furthermore, the predicted hole diameters are smaller than the actual values at low frequencies and high powers. This is because the stronger shock waves push the molten material away to the edges, which is not considered in the model.

**Table 2:** Measured and calculated data for the ablation cross-section for Al coated Si.

Power (W)	Frequency (kHz)	Averaged measured depth (μm)	Calculated depth (μm)	Accuracy (%)

5	25	2.1	2	95.2
5	50	2	2	100
5	75	1.5	1.6	93.3
10	50	2.4	2.5	95.8
15	50	3.7	4.1	89.2

At the interface of the two layers (1 μm away from the surface), the diameter of the hole can be seen suddenly decreasing in the silicon layer. This diameter change was apparent in the numerical results; however, it is not as obvious as in the experimental data. This can be explained by the results shown in Figure 3, which shows the maximum temperature at the end of the pulse on the surface and at the cross-section of the sample at 50 kHz and 5 W. In these contours, the melting points of aluminum and silicon are highlighted (660 and 1414 °C). It can be clearly seen that the molten zone in aluminum is slightly larger than silicon. Consequently, the molten aluminum is pushed away to the edges by the laser-driven shock wave. As mentioned in the previous section, the shock wave and fluid dynamics of the molten material are neglected in this model, which makes the predicted diameter reduction smaller compared to the actual profile at high pulse energies.

### Laser processing of Au-Si thin-film structure

**Figure 6:**
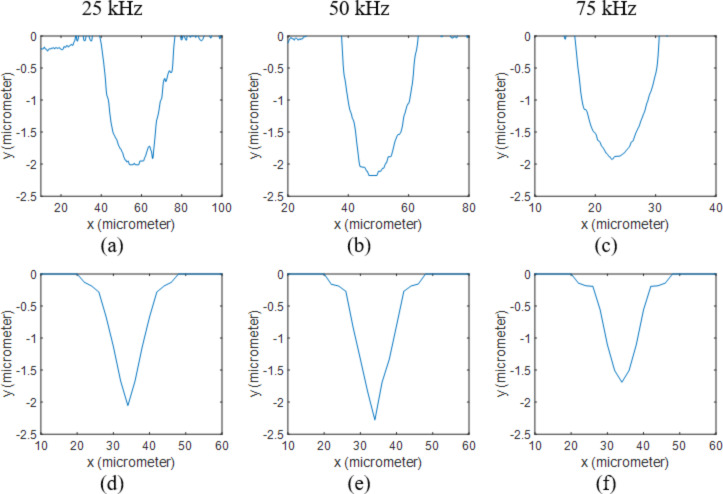
2D profiles of Au coated Si samples processed at 5 W. Results obtained from measurements when using (a) 25 kHz, (b) 50 kHz and (c) 75 kHz are compared to the ones calculated through the numerical model at (d) 25 kHz, (e) 50 kHz and (f) 75 kHz.

**Figure 7:**
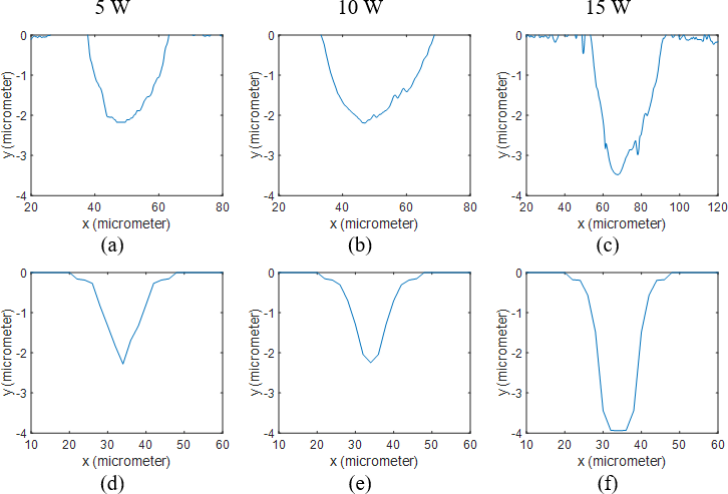
2D profiles of Au coated Si samples processed at 50 kHz. Results obtained from measurements when using (a) 5 W, (b) 10 W and (c) 15 W are compared to the ones calculated through the numerical model at (d) 5 W, (e) 10 W and (f) 15 W.

The variations of specific heat of gold with temperature are unknown. Consequently, *c*_p_ was kept constant at 129 J/kgK in the model. The melting point, boiling point, latent heat of fusion, and latent heat of vaporization are 1064 °C, 2970 °C, 64 kJ/kg and 1,736 kJ/kg respectively [22]. The calculated and measured hole profiles are shown in Figure 6 and Figure 7 and measured and calculated depths are shown in Table 3. It is evident that the holes became slightly deeper at high powers compared to bare silicon samples. This can be explained by the reasons stated in the previous section. Based on the calculated plasma absorption functions, the absorption is lower for gold-coated samples, and again, the shock wave pushed away the molten material (Figure 3), resulting in deeper and wider holes. However, the molten zone is very small for gold coated samples, which means the deeper holes created on the gold coated surface are mainly caused by the lower plasma absorption.

**Table 3:** Measured and calculated data for the ablation cross-section for Au coated Si.

Power (W)	Frequency (kHz)	Averaged measured depth (μm)	Calculated depth (μm)	Accuracy (%)

5	25	2.1	2.1	100
5	50	2.2	2.3	95.5
5	75	1.8	1.7	94.4
10	50	2.1	2.2	95.2
15	50	3.6	3.9	91.7

The results presented in this paper can be manipulated for cell patterning applications. Figure 3 depicts that, the size and shape of the heat affected zone and ablated area can be significantly altered by applying a thin film on the surface. As mentioned earlier these are the areas that become less and more biocompatible respectively. As a result, controlling the size of these areas makes the concept of patterning the cell growth by laser processing even more attractive.

## Conclusion

The model introduced in this paper is able to precisely predict the ablation depth and temperature field of the thin-film coated samples processed by a single nanosecond pulse. Silicon substrates coated with gold and aluminium thin films and pure silicon sheets were treated by single nanosecond laser pulses and experimental and numerical results were compared. This model can be extremely helpful in the fabrication of poly-Si TFTs by predicting the temperature and cooling rate during the laser processing step for controlling the grain size. Moreover, it was proved that, by applying a thin film of different materials with different thicknesses on the surface the shape and size of the heat affected zone can be controlled. The model can be further modified in future studies for laser processing of thin film coated materials along a linear pattern by introducing the scanning speed and movement of the laser beam.

## Experimental Setup

### Materials

In this paper, three different samples (wafer by University Wafer, Inc.) were processed by the laser beam at different powers and repetition rates:

1. Single crystalline silicon wafer <100>
2. c-silicon wafer <100> coated by an aluminum layer with the thickness of 1 μm.
3. c-silicon wafer <100> coated by a very thin gold layer with a thickness around 100 nm.

The thin films were deposited on the surface of the samples using physical vapor deposition technique and the samples were purchased with thin-film coating and were directly used for laser processing.

### Laser processing of thin-film structures

All the specimens were processed by an neodymium-doped yttrium aluminum garnet (Nd:YAG) nanosecond laser system (Bright Solutions SOL-20). Frequency and power can be directly controlled by changing the level of emission, whereas the pattern, line distance, and scanning speed are controlled using the EZCAD^©^ software. The wavelength of the laser beam was 1064 nm and the pulse duration was extracted from performance curves provided by the manufacturer. An iris diaphragm reduced the diameter of the output beam from 9 mm to 8 mm. The beam then went to the galvo scanner (JD2204 by Sino-Galvo), which has an input aperture of 10 mm and beam displacement of 13.4 mm. The scan lens of the galvo scanner focused the beam on the surface of the samples. The theoretical focused beam spot diameter was calculated to be around 20 µm and a Gaussian profile was assumed for the laser beam.

**Figure 8:**
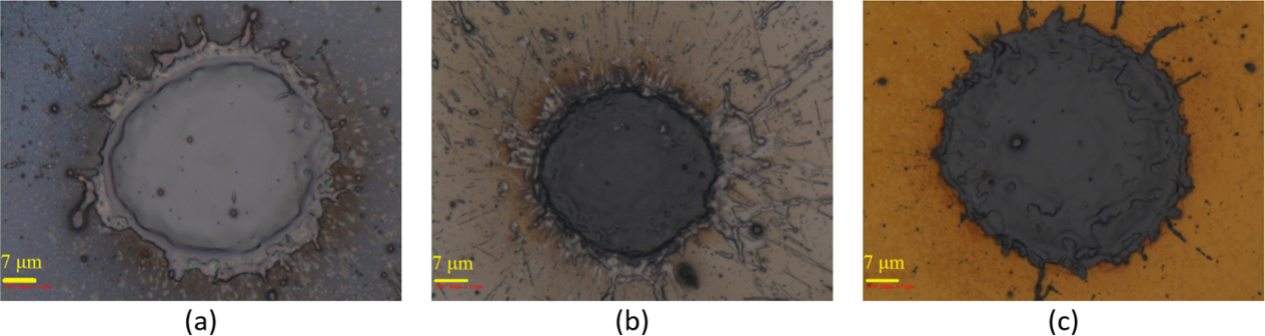
(a) Isolated pulse on the surface of silicon, (b) Isolated pulse on the surface of silicon coated by aluminum thin film, (c) Isolated pulse on the surface of silicon coated by gold thin film.

All the numerical analysis and experiments in this paper were carried out on single pulses. The laser pulses cannot be easily isolated due to limitations of the equipment. To isolate the single pulses, the scanning speed was set at its maximum value (around 1000 mm/s) and if the repetition rate was relatively low, the distance between the locations of two neighboring pulses on the surface would be long enough to remove the effect of the neighboring pulses. Therefore, a dotted pattern appears on the surface and each dot corresponds to a single pulse on the surface (Figure 8).

### Three-dimensional (3D) optical profiler

A Zeta-20 Optical Profiler (Zeta Instruments) was used to obtain surface profiles of the samples for quantitative topography measurements. This optical profiler can capture 2D and 3D images of a surface, which allows us to see the profile of the holes generated by the laser beam on the surface of the samples.

## Numerical model

### Heat transfer model

Since nanosecond pulses (where the pulse duration is in the nanosecond range) are studied in this paper, the whole process is in the hot-ablation domain. Therefore, the process can be modelled using regular laws of heat transfer and thermodynamics. A 3D transient model that was previously proven to be accurate for laser processing of the bulk materials [21] was customized for a multilayer structure and used in this paper.

In this model, the 3D heat conduction equation (Equation 1) is solved for a cubic domain considering varying thermal properties that are a function of temperature. This domain is discretized into interactive cubic cells, which can become smaller or be taken out of the system during the ablation process.

[1]
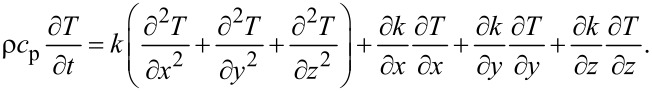


This equation was discretized and solved using the Douglas–Gunn method [23], where each time step is broken into three sub-steps, and at each sub-step, tridiagonal systems are solved implicitly in one of the spatial directions. By introducing δ^2^ operator and κ for each spatial direction (Equation 2 and Equation 3), the difference equations for each sub-step can be written as follows (Equation 4 to Equation 5):

[4]
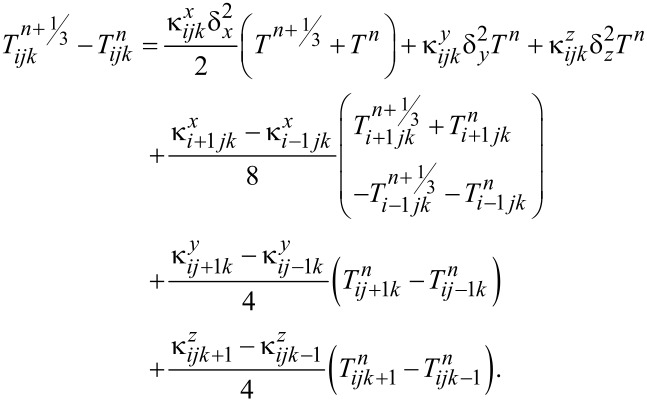


[6]
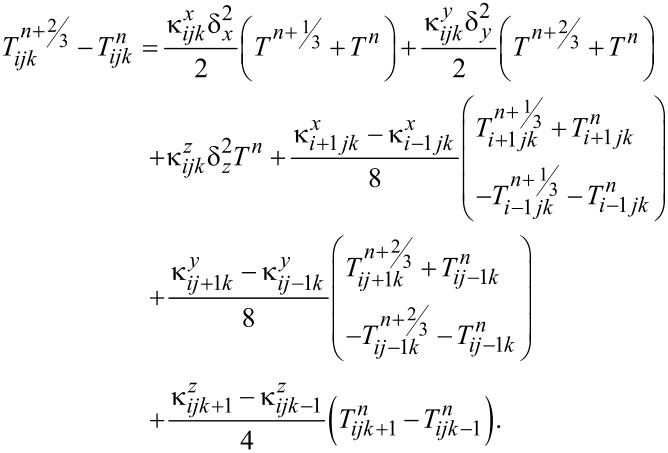


[5]
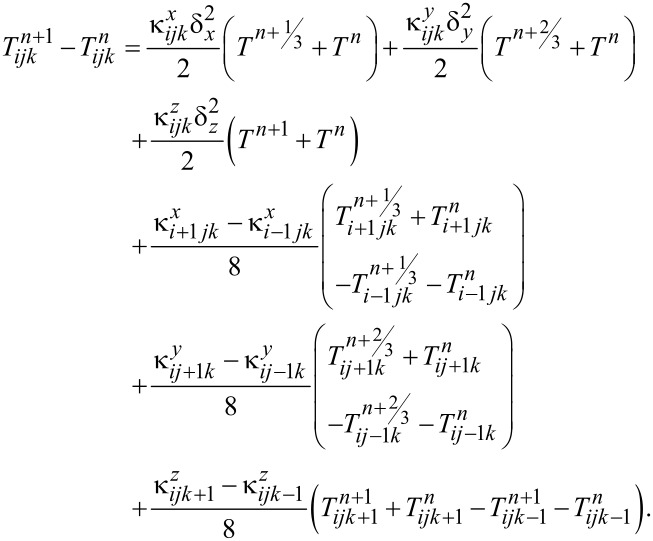


Where:

[2]
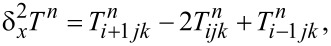


[3]
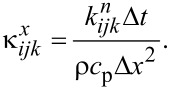


Convection, radiation and laser irradiation are the boundary conditions at the surface. The laser beam has a Gaussian profile. On the back side, insulation boundary conditions are assumed. Convection and radiation are the boundary conditions of the other sides.

**Figure 9:**
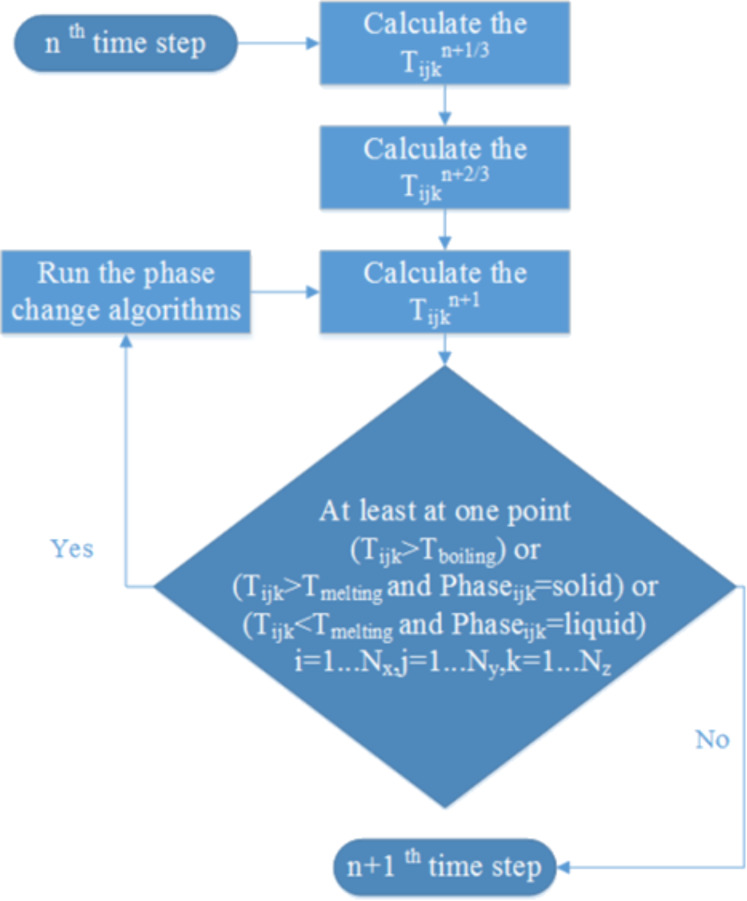
The computational flowchart at each time step.

The flowchart of the computational code is shown in Figure 9. As can be seen, after calculating the temperature at the first two sub-steps, the temperature is calculated at the last sub-step. Whenever the calculated temperature passes the melting point or boiling point, the phase change algorithms are called and the last sub-step is repeated until convergence.

### Phase change and ablation algorithms

Phase change and ablation algorithms were only applied in the last sub-step and at all the points where *T^n+^*^1/3^ or *T^n+^*^2/3^ were higher than the boiling point, they were reduced to the boiling temperature.

In the evaporation algorithm, the ablative energy (Equation 7) was calculated whenever the temperature of one or more cells was higher than the boiling temperature. In this equation, *N* is the number of cells with a temperature higher than boiling point.

[7]
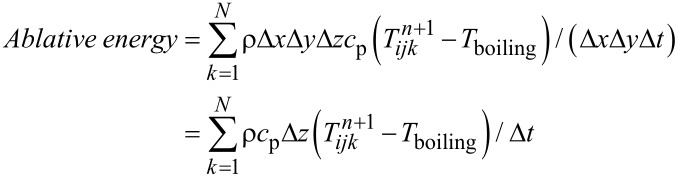


**Figure 10:**
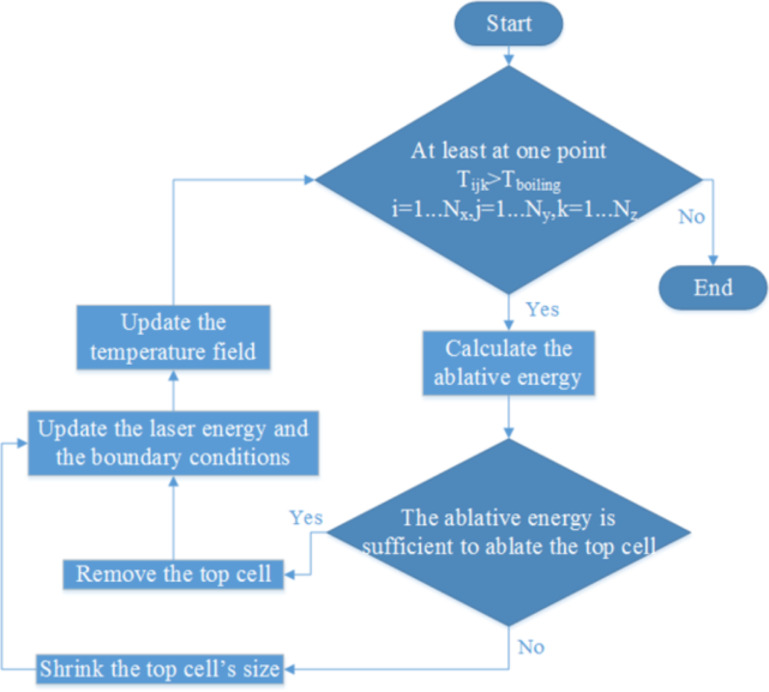
The flowchart of evaporation process.

If the ablative energy was enough to evaporate the top cell on the surface, then the cell was removed and its boundary conditions were transferred to the new top cell. Boundary conditions were updated for all the neighbouring cells and the laser energy was reduced by ρΔ*zL*_v_/Δ*t*. Otherwise, if the energy was not enough, the cell became smaller (Δ*z* was reduced by *Ablative energy* × Δ*t*/(ρ*L*_v_)) and the laser energy was decreased by the ablative energy. The last sub-step was repeated until convergence (*Ablative energy* = 0). Figure 10 shows the phase change flowchart for evaporation. Similar algorithms are considered for melting and solidification. As depicted in Figure 10, if the calculated temperature is above the boiling point, it will be determined if the whole cell should be removed or only its size must be reduced based on the described method. Then, the temperature is recalculated and this process is repeated until all the temperatures are below the boiling point.

### Plasma shielding and power loss

A plasma absorption coefficient was introduced for the plasma shielding effect (Equation 8) [21].

[8]
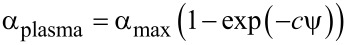


α_max_ is the maximum absorption that corresponds to the highest possible pulse power. ψ is non-dimensionalized pulse power, which is defined as the ratio of the pulse peak power to the highest possible peak power that can be generated by the laser beam. α_max_ and *c* are calculated from the experimental results.

### Nomenclature

**Table 4:** Mathematical nomenclature.

ρ	density (kg/m^3^)
*c*_p_	specific heat (J/kgK)
*T*	temperature (K)
*t*	time (s)
*k*	thermal conductivity (W/mK)
*h*	convective heat transfer coefficient (W/m^2^K)
σ	Stefan–Boltzmann constant (W/m^2^K^4^)
ε	emissivity
α	absorption
Δ*t*	time step (s)
*L*_f_	latent heat of fusion (J/kg)
*L*_v_	latent heat of vaporization (J/kg)
Λ	optical thickness
α_plasma_	plasma absorption
*h*_ablation_	ablation depth (m)
*E*_a_	energy absorbed by plasma (J)
α_max_	maximum plasma absorption
ψ	non-dimensionalized pulse power
